# Comparison between local infiltration analgesia with combined femoral and sciatic nerve block for pain management after total knee arthroplasty

**DOI:** 10.1186/s13018-020-1577-z

**Published:** 2020-02-07

**Authors:** Yi Tian, Shuai Tang, Sijin Sun, Yuelun Zhang, Lin Chen, Di Xia, Yingli Wang, Liying Ren, Yuguang Huang

**Affiliations:** 1 Department of Anesthesiology, Peking Union Medical College Hospital, CAMS&PUMC, No.1, Wangfujing, DongCheng District, Beijing, 100730 China; 2 Central Research Laboratory, Peking Union Medical College Hospital, Chinese Academy of Medical Sciences and Peking Union Medical College, No.1, Wangfujing, DongCheng District, Beijing, 100730 China; 3 Operating Room, Peking Union Medical College Hospital, Chinese Academy of Medical Sciences, Beijing, 100730 China

**Keywords:** Total knee arthroplasty, Peripheral nerve block, Local infiltration analgesia, Analgesia, Pain control

## Abstract

**Background:**

Total knee arthroplasty (TKA) is usually associated with moderate to severe postoperative pain. Peripheral nerve block (PNB) and local infiltration analgesia (LIA) are two major methods for postoperative analgesia. Femoral nerve block (FNB) leads to residual posterior knee pain; thus, currently sciatic nerve block (SNB) and LIA are two major options for supplementing FNB. However, the efficacy and safety of LIA compared with combined femoral and sciatic nerve block still remain controversial. Here, we conducted a study to analyze the postoperative analgesic efficacy of these two methods.

**Method:**

Two hundred six patients undergoing TKA were enrolled in a retrospective cohort study. The patients received either PNB or LIA. All patients in PNB group were conducted combined femoral and sciatic nerve block. All patients were encouraged to use patient-controlled analgesia (PCA) after surgery. The postoperative visual analog scale (VAS) at rest or with movement during the first 24 h and 48 h was recorded. We analyzed the VAS of 24 h, VAS of 48 h, opioid consumption, and adverse effects between PNB group and LIA group. Chi-square test and nonparametric test were used in this study.

**Results:**

There were 82 patients in the PNB group and 124 patients in the LIA group. The patients’ characteristics such as age, height, weight, and ASA showed no significant difference (*P* > 0.05). No significant differences were found (*P* > 0.05) between the two groups regarding VAS score at rest or with movement. The LIA group had less opioid consumption than the PNB group but without significant difference (*P* > 0.05). In both groups, the most common side effect was nausea, and the side effects showed no significant differences between groups (*P* > 0.05).

**Conclusion:**

Local infiltration analgesia provided a similar analgesic effect and complications compared with combined femoral and sciatic nerve block in the short term. Considering less opioid consumption with local infiltration analgesia though without significant difference and its convenience, local infiltration analgesia provided better postoperative analgesia.

## Introduction

Total knee arthroplasty (TKA) is one of the most popular treatments for chronic refractory knee pain and loss of function caused by different underlying knee disorders [1]. Total knee arthroplasty is associated with serious postoperative pain, and many patients report moderate to severe pain even past the anticipated recovery period, which is a major problem that surgeons need to address [2, 3]. Inadequate postoperative pain management can lead to acute effects, including immune system suppression, decreased mobility, and increased risk for deep vein thrombosis and pulmonary embolism [4, 5]. Most of all, poor pain management results in patient’s reluctance to mobilize the joint, hence poor functional recovery after surgery [6].

There are several methods available for postoperative analgesia including systemic opioids, continuous peripheral nerve block, peripheral nerve block, and local infiltration analgesia. Peripheral nerve block (PNB), including different techniques such as femoral nerve block, sciatic nerve block, and adductor canal block (ACB) [7], is the mainstream treatment for postoperative pain following TKA [8]. Local infiltration analgesia (LIA) was introduced to clinical practice in recent years and has been found to be helpful in relieving acute pain after TKA [9, 10]. It is performed by the surgeon at the end of the procedure and has fewer side effects of muscular weakness, offering earlier mobilization [11, 12].

Currently, both femoral nerve block (FNB) and local infiltration anesthesia (LIA) can provide effective analgesia, facilitate early mobilization, and reduce the length of hospital stay [13, 14]. Previous research has shown that some patients experience significant postoperative pain despite the use of FNB [15, 16], due to the fact that the posterior part of the knee is innervated by the sciatic nerve. Since LIA is an alternative, convenient anesthetic technique that is usually performed by orthopedic surgeons [10, 17], anesthesia via FNB combined with sciatic nerve block (SNB) and LIA are two major options for supplementing FNB to relieve pain after TKA [18, 19]. Recent studies have shown that SNB has similar anesthesia effects and opioid consumption than LIA when combined with FNB [20, 21].. However, there are few studies focusing on the comparison between LIA with combined femoral and sciatic nerve block; thus, no consensus regarding LIA versus SNB and FNB were reached. Our study aims to evaluate the analgesic effect and complication of local infiltration analgesia compared with combined femoral and sciatic nerve block after TKA. We hypothesized that local infiltration analgesia has similar analgesic effect compared with combined femoral and sciatic nerve block.

## Methods

We performed a retrospective cohort study. The Institutional Review Board (IRB) at Peking Union Medical College Hospital (PUMCH) approved this study (#S-K422). We queried hospital anesthesia records to identify all patients who were scheduled for unilateral TKA from January 2013 to December 2016. The inclusion criteria were as follows: patients for unilateral elective total knee arthroplasty, under combined femoral and sciatic nerve block or local infiltration analgesia, American Society of Anesthesiologists (ASA) classification I–III (American Society of Anesthesiologists functional status), and more than 18 years old. We excluded patients with incomplete information, who were unable to cooperate or refuse to participation, and who had an allergy to any drug administered in the study.

TKA was all performed through midline vertical incision and medial parapatellar approach by two chief surgeons who were highly experienced. All patients received general anesthesia during surgery with standard drugs. In the PNB group, patients preoperatively received ultrasound-guided combined femoral and sciatic block by two anesthesiologists. 0.5% plain ropivacaine was injected to the desired sonographic anatomical location. In the LIA group, patients received 50 ml of cocktail mixture containing 30 ml ropivacaine (10 mg/ml), 0.5 ml morphine (10 mg/ml), 1 ml diprospan (5 mg/ml), and normal saline to make up to 50 ml. This solution was infiltrated into the joint capsule in particular the posterior capsule, retinacular tissue, subcutaneous tissues, and anterior fat pad. Patients in PNB group received no injection. The LIA procedure was conducted by two chief surgeons after the main step of the surgery.

After surgery, all patients received a standard postoperative regimen of parecoxib (Dynastat®, Pfizer) 40 mg bid for 3 days as well as an intravenous patient-controlled analgesia (PCA) pump for 48 h. PCA pump was morphine 40–60 mg in normal saline 250 ml, which was programmed to give a background dose of 0–4 ml/h, a 3–4-ml bolus on demand, a lock-out time of 10–15 min, and a maximum dose of 40–60 ml/4 h. All patients were encouraged to use PCA as often as needed. The PCA record of each patient was monitored and administered in the Department of Anesthesiology which can be searched through intranet or the medical record.

### Outcomes: pain scores

All patients were educated preoperatively by the Acute Pain Service team about pain assessment using a visual analog scale (VAS): 0 = no pain and 10 = worst pain imaginable. VAS scores at rest or with movement during the first 24 h and 48 h once a day were recorded by the specialists of Acute Pain Service team [22] before physical therapy during hospitalization. No data were imputed for the primary outcome if the patient was asleep or unable to report VAS.

### Side effects

Complications including nausea and vomiting, pruritus, and sedation were recorded. The VAS score and complications were administered in the department of anesthesiology which can be searched through intranet. And it was also documented in the medical record of each patient.

### Statistical analysis

We described the baseline characteristics of patients in the PNB and LIA groups in tabular form. The difference of baseline characteristics between the PNB and LIA groups were compared, and factors with *P* value less than 0.1 were regarded as potential confounders. Based on the non-normally distribution of the main outcomes including VAS on rest, VAS with movement, and PCA consumption, they were compared using Mann-Whitney test between the PNB and LIA groups. If there were unbalanced baseline characteristics between groups, the main outcomes were further analyzed stratified by the confounders. Side effects in the PNB and LIA groups were compared using the chi-squared test. As sex was an important impact factor for postoperative nausea and vomiting (PONV), the difference of the side effects between groups was further striated and adjusted using logistic regression by sex. A two-side *P* value less than 0.05 was considered statistically significant. Statistical analyses were done in the STATA software (Version 14.1[StataCorp., 4905 Lakeway College Station, TX 77845, USA]).

## Results

A total of 254 patients were reviewed using the electronic medical records system. There were 46 patients in the PNB group and 2 patients in the LIA group who received continuous peripheral nerve block, which were excluded. Finally, 206 patients were included for analysis. For the PNB group, 82 patients were identified, all of which received combined femoral and sciatic nerve block. For the LIA group, 124 patients were identified. Patient demographics are shown in Table 1. There are no statistical differences between the two groups.

**Table 1:** Patient demographics

Items	PNB (*N* = 82)	LIA (*N* = 124)	*P* value
Gender			0.10
Male	16 (19.51%)	37 (29.84%)	
Female	66 (80.49%)	87 (70.16%)	
Age	67.1 (9.4)	65.8 (12.6)	0.40
Height	161.5 (6.4)	162.0 (8.4)	0.70
Weight	68.8 (8.9)	69.4 (12.0)	0.70
BMI	26.4 (3.3)	26.5 (4.3)	0.85
ASA			0.57
I	6 (7.32%)	14 (11.29%)	
II	66 (80.49%)	98 (79.03%)	
III	10 (12.20%)	12 (9.68%)	

Baseline characteristics presented as number (%) or mean (standard deviation)

*PNB* peripheral nerve block, *LIA* local infiltration analgesia

**Fig. 1:**
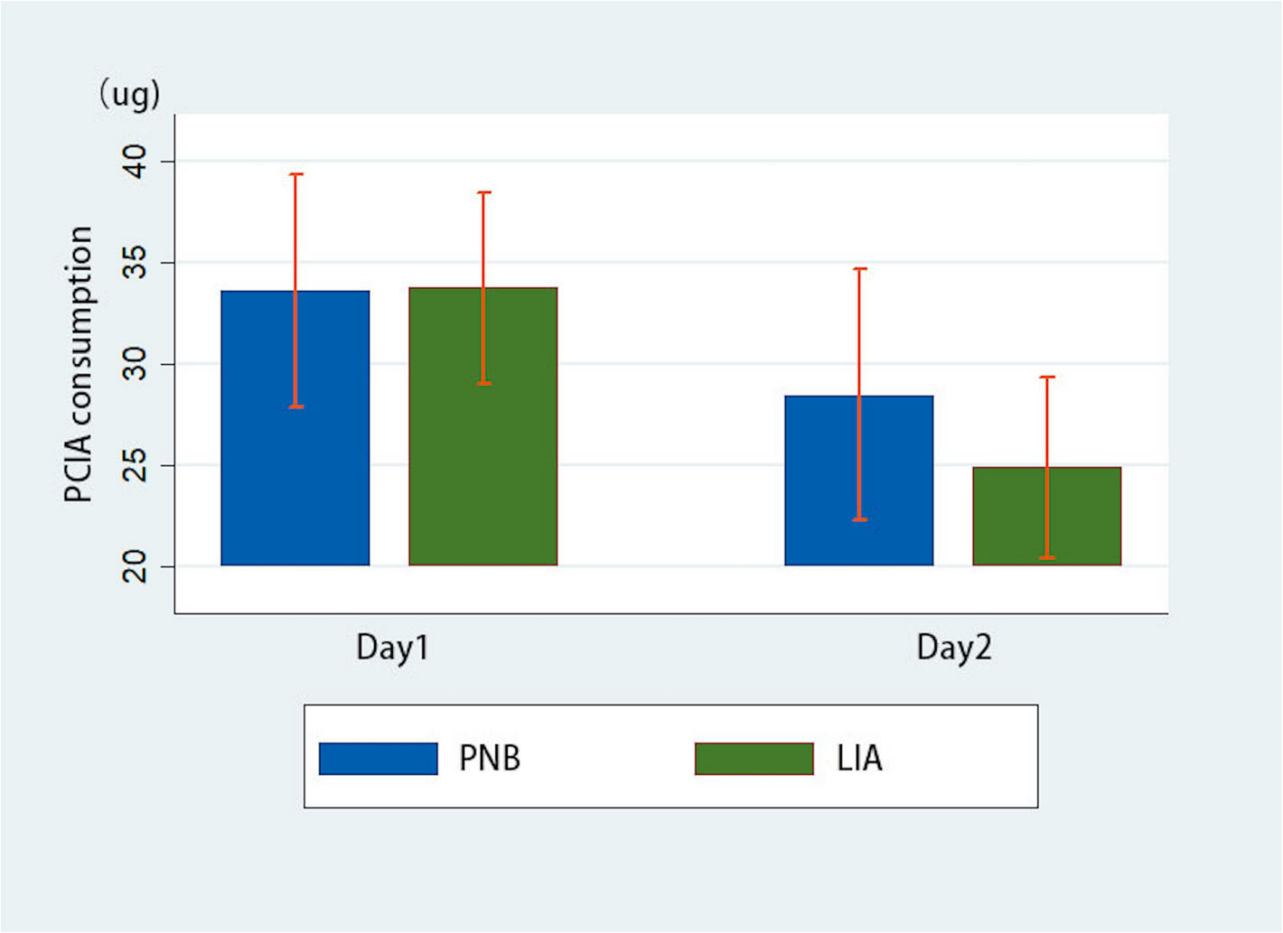
Trend of opioids consumption postoperative between groups

Regarding pain management, there was no significant difference (*P* > 0.05) between the two groups on VAS score at rest or with movement during the first 24 h and 48 h (Table 2). The LIA group had less PCA consumption than the PNB group on POD1 (33.74 vs. 33.62, *P* = 0.86) and POD2 (24.88 vs. 28.45, *P* = 0.55), but without significant difference (Table 2). The trend of opioids consumption is shown in Fig. 1.

**Table 2:** VAS and PCA of the two groups

Items	PNB (*N* = 82)	LIA (*N* = 124)	*P* value
Day 1 VAS on rest	1.35	1.35	0.95
Day 2 VAS on rest	0.73	0.85	0.42
Day 1 VAS with movement	3.25	3.16	0.82
Day 2 VAS with movement	2.55	2.47	0.80
Day 1 PCA consumption	33.62	33.74	0.86
Day 2 PCA consumption	28.45	24.88	0.55

Outcomes: VAS on rest, VAS with movement, and PCA consumption presented as mean

*PNB* peripheral nerve block, *LIA* local infiltration analgesia, *VAS* visual analog scale, *PCA* patient-controlled analgesia

In both groups, nausea was the most common side effect on POD1 and POD2 (13–31%), followed by vomiting (2–18%), sedation (3–13%), and pruritus (1–3%). The side effects including nausea, vomiting, pruritus, and sedation have similar occurrence rates. Notably, nausea and vomiting on day 2 were significantly more common in the PNB group compared with the LIA group (*P* = 0.02). However, after controlling for or being striated by gender between these two groups, which is a well-known risk factor for nausea and vomiting [23], the difference was not statistically significant. The details of the side effects are presented in Table 3.

**Table 3:** Details of the side effects

Items	Total			Male			Female		
	PNB	LIA	*P* value	PNB	LIA	*P* value	PNB	LIA	*P* value
Day 1 nausea			0.07			0.59			0.16
Yes	26 (32%)	25 (20%)		2 (17%)	4 (11%)		24 (34%)	21 (25%)	
No	56 (68%)	99 (80%)		10 (73%)	33 (89%)		46 (66%)	66 (75%)	
Day 1 vomit			0.08			0.43			0.31
Yes	14 (18%)	13 (10%)		1 (8%)	1 (3%)		13 (20%)	12 (14%)	
No	64 (82%)	111 (90%)		12 (92%)	36 (97%)		52 (80%)	75 (86%)	
Day 1 pruritus			0.90			0.54			0.69
Yes	2 (3%)	3 (3%)		0 (0%)	1 (3%)		2 (4%)	2 (2%)	
No	68 (97%)	117 (97%)		13 (100%)	35 (97%)		55 (96%)	82 (98%)	
Day 1 sedation			0.20			0.86			0.22
Yes	10 (13%)	9 (7%)		1 (7%)	2 (5%)		9 (14%)	7 (8%)	
No	68 (87%)	115 (93%)		14 (93%)	35 (95%)		54 (86%)	80 (92%)	
Day 2 nausea			0.02			0.12			0.09
Yes	21 (26%)	16 (13%)		3 (25%)	3 (8%)		18 (26%)	13 (15%)	
No	61 (74%)	108 (87%)		9 (75%)	34 (92%)		52 (74%)	74 (75%)	
Day 2 vomit			0.05			0.13			0.91
Yes	6 (8%)	12 (10%)		2 (13%)	2 (5%)		4 (6%)	10 (11%)	
No	71 (92%)	112 (90%)		13 (87%)	35 (95%)		58 (74%)	77 (89%)	
Day 2 pruritus			0.77			–			0.85
Yes	1 (1%)	1 (0%)		0 (0%)	0 (0%)		1 (2%)	1 (1%)	
No	69 (99%)	105 (100%)		13 (100%)	35 (100%)		56 (98%)	73 (99%)	
Day 2 sedation			0.20			0.85			0.10
Yes	5 (6%)	3 (3%)		1 (7%)	2 (6%)		4 (6%)	1 (1%)	
No	72 (94%)	111 (97%)		13 (93%)	33 (94%)		58 (94%)	78 (99%)	

Side effects on POD1and 2 including nausea, vomiting, urinary retention, pruritus, and sedation presented as number (%)

*PNB* peripheral nerve block, *LIA* local infiltration analgesia

## Discussion

The present study was aimed to compare the effects and safety of PNB and LIA. After analyzing VAS, morphine consumptions, and side effects, the results indicate that LIA has similar postoperative analgesic efficacy and complications than combined femoral and sciatic nerve block. Thus, we believe that LIA is as effective and safe as PNB.

Adequate pain relief after TKA is important as it enhances rehabilitation [24, 25], which is of key importance for a satisfactory outcome. In our study, postoperative pain is managed in a multimodal style including PNB or LIA followed by intravenous PCA within the first 2 days after surgery. VAS both at rest and with movement was evaluated. Postoperative VAS remained at a low level in both groups; thus, sufficient postoperative analgesia was achieved with either PNB or LIA technique. We found no significant difference between the two groups on VAS at rest or with movement during the first 24 h and 48 h, which indicates that the pain relief effect of both methods is comparable. The reason for this is due to the multimodal analgesia including PCA pump and LIA which make patients in an acceptable range of pain, thus resulting in a similar degree of pain relief. As for all kinds of peripheral nerve block, LIA has similar pain relief compared with single femoral nerve block [26, 27], continuous femoral nerve block [27, 28], and single sciatic nerve block [18, 20]. However, currently, few studies focus on the comparison between local infiltration analgesia with combined femoral and sciatic nerve block and do not reach a consensus. One of them has the same results as pain relief was similar between the two groups [29, 30]; other studies indicate that FNB combined with SNB provides superior pain relief than LIA [31, 32]. Therefore, this study enriches existing literatures in this field of comparing combined femoral and sciatic nerve block.

Although the pain scores were similar in the two groups, the LIA group (24.88 mg) had less PCA consumption than the PNB group (28.45 mg) up to 48 h postoperatively. Opioid consumption is considered an objective method of measuring pain. The tendency of lower efficacy with femoral and sciatic block may be due to the fact that some part of the knee is innervated by the other nerves such as the obturator nerve [33, 34] and some cutaneous nerve [35] which are still not blocked. Thus, a peripheral nerve block may need supplementary treatment with more systemic analgesics such as opioids and NSAIDs [32]. Another explanation why LIA is more effective might be the better effect of nerve block of the intraarticular drugs [36]. Analgesic effect of NSAIDs is better after intraarticular administration than after systemic IV injection [37]. The reduction in PCA consumption from POD1 to POD2 is larger in the LIA group (8.86 mg) compared with the PNB group (5.17 mg), which indicates that the effect of peripheral nerve block may be shorter than LIA. It can be interpreted by the long-lasting anti-inflammatory effect of diprospan locally and systemically, which were confirmed in the past study [38].

The incidence of side effects was similar between the two groups. Opioid-related adverse effects such as postoperative nausea and vomiting, antiemetic use, and postoperative sedation/drowsiness were reported in previous studies [39, 40], but we did not find significant differences between the two groups in this study. This could be because the difference between the two groups in PCA consumption was not large enough to cause sufficient differences in side effects. It is possible that side effects of opioids are dose dependent [40] and that when larger doses are administered, the incidence of side effects increases and then becomes more clinically significant.

LIA is a relatively safe operation. LIA is performed by injecting analgesic drugs into the soft tissues around the surgical site including both the anterior and posterior knee capsules [41]. There were no previous literatures reporting medical complications of LIA because there are no large blood vessels and nerves in the surgery area.

The limitation of this study comes from the retrospective design. Our study is a single-center clinical trial, and selective bias is inevitable. Second, due to the limitation of study design, we cannot compare functional outcomes and long-term outcomes between groups. Nikolajsen et al. reported that more than 12% of patients had moderate to severe postoperative pain even more than a year after operation [42]. Even more, more than twice as many patients have chronic pain after revision TKA surgery compared with primary TKA [43]. But our study focused on the short-term outcome at 48 h after surgery instead of chronic pain. Therefore, multicenter studies focusing on functional outcome and long-term pain management are needed in the future.

In conclusion, local infiltration analgesia provided a similar analgesic effect and complications compared with combined femoral and sciatic nerve block in the short term. LIA is a relatively convenient and easy method compared with nerve block, which can be administered without the need for specialist additional equipment. Thus, LIA should be considered as a viable and safe alternative to combined femoral and sciatic nerve block for early pain relief following TKA, especially in developing countries where LIA has not been widely applied.

## Data Availability

The datasets generated and analyzed during the current study are available from the corresponding author on reasonable request.

## Abbreviations

ACB: Adductor canal block
ASA: American Society of Anesthesiologists
LIA: Local infiltration analgesia
PCA: Patient-controlled analgesia
PNB: Peripheral nerve block
TKA: Total knee arthroplasty
VAS: Visual analog scale
